# Association Between Physical Activity and Risk of Disabling Dementia in Japan

**DOI:** 10.1001/jamanetworkopen.2022.4590

**Published:** 2022-03-29

**Authors:** Hikaru Ihira, Norie Sawada, Manami Inoue, Nobufumi Yasuda, Kazumasa Yamagishi, Hadrien Charvat, Motoki Iwasaki, Shoichiro Tsugane

**Affiliations:** 1 Epidemiology and Prevention Group, Center for Public Health Sciences, National Cancer Center, Tokyo, Japan; 2 Department of Public Health, Kochi University Medical School, Kochi, Japan; 3 Health Services Research and Development Center, Department of Public Health Medicine, Faculty of Medicine, University of Tsukuba, Tsukuba, Japan; 4 National Institute of Health and Nutrition, National Institutes of Biomedical Innovation, Health and Nutrition, Tokyo, Japan

## Abstract

**Importance:**

The associations of daily total physical activity and total moderate to vigorous physical activity (MVPA) with dementia are still unclear.

**Objective:**

To investigate the association between daily total physical activity and subsequent risk of disabling dementia in large-scale, extended follow-up prospective study.

**Design, Setting, and Participants:**

This prospective cohort study used data from questionnaires collected between 2000 and 2003 from 8 areas from the Japan Public Health Center-based Prospective Disabling Dementia Study. Participants included adults aged 50 to 79 years in with available follow-up data on disabling dementia. Data analysis was performed from February 1, 2019, to July 31, 2021.

**Exposures:**

Daily total physical activity, total MVPA, and leisure-time MVPA.

**Main Outcomes and Measures:**

The main outcome was incidence of disabling dementia during the dementia ascertainment period between 2006 and 2016, based on the national long-term care insurance system. Risks of dementia in association with daily total physical activity, total MVPA, and leisure time MVPA were calculated using multivariable adjusted hazard ratios (aHRs).

**Results:**

Among 43 896 participants (mean [SD] age, 61.0 [7.5] years; 23 659 [53.9%] women), 5010 participants were newly diagnosed with disabling dementia during a mean (SD) of 9.5 (2.8) years in the dementia ascertainment period. In the highest daily total physical activity group, compared with the lowest activity group, risk of dementia was lower in men (aHR, 0.75 [95% CI, 0.66-0.85]; *P* for trend < .001) and women (aHR, 0.75 [95% CI, 0.67-0.84]; *P* for trend < .001). Similar inverse associations were observed in men and women for total MVPA (men: aHR, 0.74 [95% CI, 0.65-0.84]; *P* for trend < .001; women: aHR, 0.74 [95% CI, 0.66-0.83]; *P* for trend < .001) and leisure-time MVPA (men: aHR, 0.59 [95% CI, 0.53-0.67]; *P* for trend < .001; women: aHR, 0.70 [95% CI, 0.63-0.78]; *P* for trend < .001). However, these inverse associations disappeared when participants diagnosed with disabling dementia within 7 years of the starting point were excluded in men (aHR, 0.93 [95%CI, 0.77-1.12]) and within 8 years were excluded in women (aHR, 0.86 [95%CI, 0.71-1.04]). The association remained significant among men in the highest vs lowest group of leisure-time MVPA, after excluding participants diagnosed within the first 9 years (aHR, 0.72 [95% CI, 0.56-0.92]; *P* for trend = .004).

**Conclusions and Relevance:**

This cohort study examined associations of daily total physical activity and total MVPA with risk of disabling dementia. The findings suggest that a high level of leisure-time MVPA was associated with decreased risk of disabling dementia in men.

## Key Points

**Question:**

What are the associations of daily total physical activity, total moderate-to-vigorous physical activity (MVPA), and leisure-time MVPA with risk of dementia in the general population in Japan?

**Findings:**

This cohort study including 43 896 participants investigated associations of daily total physical activity and total MVPA with risk of disabling dementia. However, a higher level of leisure-time MVPA was statistically significantly associated with decreased risk of disabling dementia in men.

**Meaning:**

These findings suggest that higher leisure-time MVPA may be associated with the prevention of disabling dementia in men.

## Teaser

This cohort study assesses the association between physical activity and risk of disabling dementia in Japan.

## Introduction

Dementia is one of the major causes of disability and dependency among older people. Approximately 50 million people worldwide currently have dementia, and nearly 10 million new cases are diagnosed every year. The World Health Organization has listed dementia as a public health priority.^1^

Physical activity is a potential preventive factor for dementia^1^ and has been shown to have an inverse association with dementia incidence in several epidemiological studies.^2,3,4,5,6,7,8,9,10^ However, these studies were conducted with short-term follow-up periods. In contrast, some cohort studies^11,12,13^ with long-term follow-up reported no association between leisure-time physical activity and risk of dementia and suggested that the observed inverse association between them may be attributable to a reverse causation bias, defined in epidemiology as when exposure to a disease process is reversed.^14^ In dementia studies, for example, a decline in physical activity may not be the cause of dementia, but rather a consequence arising in the preclinical phase of dementia. However, because the preclinical phase of dementia is relatively long, studies with shorter follow-up are unable to identify this association.

Similarly, a meta-analysis that included 1300 incident cases of all-cause dementia occurring after 10 years from the start of follow-up also found a null association between leisure-time physical activity and dementia, and suggested a reverse causation bias.^15^ In contrast, although daily total physical activity, including leisure-time physical activity and nonexercise physical activity, contributes to total daily energy expenditure and thereby also confers important health benefits,^16,17^ only a few epidemiological studies^3,8,9^ have focused on the association between daily total physical activity and risk of dementia. These studies reported an association between a high level of daily total physical activity and risk of dementia but did not consider the possibility of reverse causation bias because of their short follow-up. In addition, it remains unclear whether the association of daily total moderate to vigorous physical activity (MVPA) with incident dementia may have a potential reverse causation bias, although many studies report that MVPA is beneficial for dementia^3,4^ and brain health.^18,19,20,21^ In this study, to investigate whether daily total physical activity and MVPA in daily total time and in leisure-time are associated with the subsequent risk of dementia, and whether the associations may be subject to reverse causation bias, we analyzed a large number of adults with disabling dementia, as certified under Japan’s national long-term care insurance (LTCI) system within a large prospective cohort study with long-term follow-up.

## Methods

This cohort study was approved by the institutional review board of the National Cancer Center, Tokyo, Japan. Study participants were informed of the purpose of the study, and those completing the survey questionnaire were regarded as consenting to participation. This study followed the Strengthening the Reporting of Observational Studies in Epidemiology (STROBE) reporting guideline.

### Study Population

The Japan Public Health Center-based Prospective Study (JPHC Study) was launched in 1990 for cohort I and in 1993 for cohort II. Details of the study are reported elsewhere.^22^ Cohort I included residents aged 40 to 59 years in 5 public health center (PHC) areas (Iwate, Akita, Nagano, Okinawa, and Tokyo) and cohort II included residents aged 40 to 69 years in 6 PHC areas (Ibaraki, Niigata, Kochi, Nagasaki, Okinawa, and Osaka). The 2 cohorts included a total of 140 420 men and women. Data analysis was performed from February 1, 2019, to July 31, 2021.

In the JPHC Disabling Dementia Study, among 140 420 total participants, we included 62 401 participants in 8 PHC areas with available follow-up data on disabling dementia: Omonogawa and Yokote districts in Yokote in Akita Prefecture; Iwase district in Sakuragawa and Tomobe district in Kasama in Ibaraki Prefecture; Usuda district in Saku in Nagano Prefecture; Kagami and Noichi districts in Konan in Kochi Prefecture; and Gushikawa district in Uruma in Okinawa Prefecture. Of 62 401 participants, we excluded 134 participants who were ineligible (non-Japanese nationality, not present from baseline, incorrect birth data, or duplicate enrollment) and 11 591 participants who moved away or died before the starting point for follow-up of case ascertainment, leaving 50 676 participants for analysis. Among these 50 676, 45 043 responded to the 10-year follow-up questionnaire (response rate, 88.9%), of whom 472 with a severe physical limitation who had difficulty going out independently and 675 with missing information on physical activity were excluded (eFigure in the Supplement).

### Follow-up and Identification of Disabling Dementia

We used certification records in the national LTCI system to identify study participants with disabling dementia. Criteria were the same as those used in previous studies in Japan.^23,24^ The LTCI system is a compulsory insurance system introduced by the Ministry of Health, Labor and Welfare of Japan in 2000 that is administered by municipalities.^25^ Residents aged 65 years and older and those with disability aged 40 to 64 years wishing to receive long-term care services apply as functionally disabled with the municipality. The municipal government assesses the applicant’s functional health status by a comprehensive assessment and obtains a primary care physician’s written opinion of the disability. A physician completes the dementia rating scale according to the manual issued by the national government. We defined disabling dementia as certification at any level of needed long-term care, within the range of severity of cognitive disability (grade IIa, IIb, IIIa, IIIb, IV, or M) on the dementia rating scale derived from a primary care physician’s written opinion. Regarding validation of the dementia rating scale, a 2009 study^26^ reported that this is well correlated with the Mini-Mental State Examination score (*r* = −0.74).

Because certification records in the LTCI system were available from 2006, the starting point was defined as January 1, 2006. Records of participants in the system were collected during the dementia ascertainment period from the starting point until December 31, 2016.

### Assessment of Physical Activity

This study consisted of a baseline survey (survey 1), and follow-up surveys at 5 years (survey 2) and 10 years (survey 3). The questionnaire item on physical activity item differed in each of the 3 surveys. We assessed physical activity as the main exposure variable using the 10-year follow-up survey conducted in 2000 to 2003, because it included more comprehensive information on physical activity than the other 2 and allowed evaluation of daily total MVPA and leisure-time MVPA in the 10-year follow-up survey only. Participants were asked the number of hours spent at sitting, standing, walking, and strenuous work in nonleisure time on a typical day in the last year, and the frequency and number of hours spent walking slowly, such as when taking a walk; walking quickly; light to moderate exercise, such as in golf or gardening; and strenuous exercise, such as tennis, jogging, aerobics, or swimming, in leisure time. We assigned metabolic equivalents (METs)^27^ as 1.3 METs for sitting, 2.0 METs for standing, 3.0 METs for walking, and 6.0 METs for strenuous work in nonleisure time. For leisure time, we assigned 2.8 METs for walking slowly, 4.0 METS for walking quickly, 3.0 METs for light to moderate exercise, and 6.0 METs for strenuous exercise.^27^ We assigned 0.9 METs for sleep and 1.3 METs for other activities. Finally, we calculated daily total physical activity by the sum of lengths of time spent for the respective activities multiplied by the assigned METs. If the total time spent in the respective activities exceeded 24 hours, hourly METs were first calculated by dividing the sum of METs by the total time spent, and then converted to daily METs by multiplying the number obtained by 24. Further, we calculated total MVPA and leisure-time MVPA by considering activities with an intensity of 3.0 METs or higher. Spearman rank correlation coefficient for daily total physical activity and 24-hour activity record was 0.672, and for total MVPA and 24-hour activity record was 0.610.^28^

### Statistical Analysis

Person-years of the dementia ascertainment period were calculated for each participant from the starting point until the date of disabling dementia diagnosis, date of migration from a study area to a nonstudy area, date of death, or end of follow-up (December 31, 2016), whichever occurred first.

Adjusted hazard ratios (aHRs) and 95% CIs for disabling dementia according to physical activity, including daily total physical activity, total MVPA and leisure-time MVPA, were calculated using Cox proportional hazard regression models in men and women. The basic models stratified by area (8 city-level municipalities) and were adjusted for age (continuous). Multivariable models included covariates in the same questionnaire with primary exposure at the 10-year follow-up survey, such as smoking status (never, former, 1 to 19 cigarettes/d, ≥20 cigarettes/d), alcohol intake status (nondrinker or occasional drinker, 1 to <150 g/wk, 150 to <300 g/wk, ≥300 g/wk), body mass index (BMI; calculated as weight in kilograms divided by height in meters squared and categorized as <18.5, 18.5-24.9, 25-29, and ≥30), past history of diabetes (yes or no), use of medication for hypertension (yes or no), and occupation (primary industry, secondary or tertiary industry, unemployed, or household duties). These covariates are collected at same time with primary exposure. Linear trends were assessed by assigning ordinal numbers to categories of physical activity. Furthermore, we performed analyses after sequential exclusion of incident disabling dementia arising each year from the first year of the dementia ascertainment period to assess for potential reverse causation bias owing to a decline in physical activity level before the incidence of dementia. Additionally, we performed 4 sensitivity analyses. First, we performed the same analyses in participants aged 65 years and older. Second, we analyzed competing risk using the Fine and Gray subdistribution hazards model,^29^ on the basis that death (during 2006 to 2016) before disabling dementia incidence can be a competing event. Third, we analyzed the association between the risk of disabling dementia and change in daily total physical activity between the 5-year and 10-year questionnaires by considering changes between categories as determined by tertile of distribution of daily total physical activity, albeit that we did not assess total MVPA and leisure-time MVPA using the 5-year questionnaire because of the simplicity of its questions. Fourth, we also conducted a subgroup analysis in a multivariable model with the addition of education level (junior high school or higher education) to covariates in cohort I that included information on education. Multiple imputations were performed for missing covariate values using the full-conditional specification method with arbitrary missing patterns, creating 10 imputed data sets. All *P* values were 2-sided, and significance level was set at *P* < .05. All statistical analyses were performed with SAS software versions 9.3 and 9.4 (SAS Institute).

## Results

The final cohort included 43 896 participants (mean [SD] age, 61.0 [7.5] years; 23 659 [53.9%] women) during 417 027 person-years of follow-up, and 5010 participants (11.4%) were newly diagnosed with disabling dementia during a mean (SD) dementia ascertainment period of 9.5 (2.8) years (eFigure in the Supplement). During the dementia ascertainment period, 11 077 participants (21.9%) died, 2287 participants (4.5%) moved away, and 6 participants (0.01%) were lost to follow-up before incident disabling dementia. Participant characteristics at the 10-year follow-up survey by daily total physical activity in men and women are shown in Table 1. In both sexes, participants with a high daily total physical activity level were younger, had a lower BMI, higher proportion of never smoking, higher proportion of drinking, lower proportion of unemployment, and lower prevalence of diabetes and hypertension.

**Table 1.** Characteristics of Participants According to Daily Total Physical Activity in Men and Women

Characteristic	Physical activity quartile, men, No. (%)				*P* value	Physical activity quartile, women, No. (%)				*P* value
	1	2	3	4		1	2	3	4	
No.	5059	5059	5059	5060		5914	5915	5915	5915	
Age at survey, mean (SD), y	62.6 (8.0)	60.1 (7.3)	60.4 (7.0)	59.6 (6.8)	<.001	64.4 (8.1)	61.3 (7.5)	60.2 (6.9)	59.3 (6.7)	<.001
BMI										
<18.5	185 (3.8)	144 (2.9)	128 (2.6)	133 (2.7)	.009	274 (4.9)	240 (4.1)	212 (3.6)	203 (3.5)	<.001
18.5-24.9	3243 (66.2)	3355 (66.9)	3383 (67.6)	3528 (70.8)		3491 (62.0)	3889 (66.7)	3981 (68.1)	3932 (67.5)	
25.0-29.9	1346 (27.5)	1411 (28.1)	1380 (27.6)	1228 (24.6)		1582 (28.1)	1517 (26.0)	1489 (25.5)	1507 (25.9)	
≥30	127 (2.6)	106 (2.1)	113 (2.3)	96 (1.9)		282 (5.0)	185 (3.2)	163 (2.8)	185 (3.2)	
Smoking status										
Never	1189 (24.2)	1385 (27.6)	1363 (27.2)	1369 (27.4)	.001	5278 (93.3)	5440 (93.3)	5436 (93.3)	5380 (92.4)	.02
Past	1729 (35.2)	1658 (33.0)	1643 (32.8)	1333 (26.7)		106 (1.9)	103 (1.8)	104 (1.8)	82 (1.4)	
1-19 cigarettes/d	654 (13.3)	567 (11.3)	582 (11.6)	654 (13.1)		182 (3.2)	176 (3.0)	189 (3.2)	239 (4.1)	
≥20 cigarettes/d	1340 (27.3)	1408 (28.1)	1416 (28.3)	1634 (32.8)		93 (1.6)	110 (1.9)	96 (1.7)	121 (2.1)	
Alcohol intake										
None or occasional	1128 (22.9)	905 (18.2)	877 (17.6)	932 (18.7)	<.001	4540 (81.3)	4357 (74.8)	4285 (73.6)	4218 (72.9)	<.001
1 to <150 ethanol g/week	1571 (31.9)	1601 (32.1)	1485 (29.9)	1293 (25.9)		864 (15.5)	1234 (21.2)	1300 (22.3)	1312 (22.7)	
15 to <300 ethanol g/week	877 (17.8)	1020 (20.5)	1031 (20.7)	945 (19.0)		104 (1.9)	154 (2.6)	155 (2.7)	170 (2.9)	
≥300 ethanol g/week	1352 (27.4)	1458 (29.3)	1578 (31.7)	1814 (36.4)		74 (1.3)	84 (1.4)	84 (1.4)	88 (1.5)	
Occupation										
Primary industry	529 (12.2)	512 (11.0)	1060 (23.3)	1345 (31.1)	<.001	644 (12.3)	634 (11.3)	886 (15.8)	1584 (28.9)	<.001
Secondary or tertiary industry	2350 (54.3)	3212 (69.1)	2864 (62.8)	2717 (62.7)		1359 (25.9)	2045 (36.4)	2249 (40.1)	2572 (46.9)	
Household duty	NA	NA	NA	NA		2103 (40.1)	2395 (42.6)	2105 (37.5)	1107 (20.2)	
Unemployed	1452 (33.5)	926 (19.9)	636 (14.0)	269 (6.2)		1136 (21.7)	552 (9.8)	371 (6.6)	223 (4.1)	
Disease history										
Diabetes	532 (10.5)	457 (9.0)	414 (8.2)	359 (7.1)	<.001	371 (6.3)	302 (5.1)	264 (4.5)	254 (4.3)	<.001
Hypertension	1512 (29.9)	1241 (24.5)	1143 (22.6)	1030 (20.4)	<.001	1966 (33.2)	1576 (26.6)	1391 (23.5)	1193 (20.2)	<.001
Physical activity, mean (SD), MET-h/d										
Daily total physical activity	29.6 (1.5)	34.6 (1.5)	42.6 (3.3)	60.5 (7.4)	<.001	29.8 (1.6)	34.5 (1.3)	40.1 (2.3)	55.0 (8.2)	<.001
Total MVPA	1.4 (1.6)	6.4 (3.1)	17.0 (5.5)	44.8 (15.7)	<.001	1.3 (1.6)	5.9 (2.9)	13.2 (4.4)	36.3 (16.0)	<.001
Leisure-time MVPA	0.5 (1.0)	1.4 (2.1)	1.9 (3.0)	1.7 (4.3)	<.001	0.4 (0.8)	1.0 (1.6)	1.7 (2.4)	2.0 (3.8)	<.001

Abbreviations: BMI, body mass index; MET, metabolic equivalent; MVPA, moderate to vigorous physical activity; NA, not applicable.

Table 2 shows that the higher level of daily total physical activity was inversely associated with disabling dementia risk in both sexes. Compared with participants in the first quarter (Q1) of daily total physical activity, risk of dementia decreased with increasing physical activity for men (Q2: aHR, 0.73 [95% CI, 0.65-0.82]; Q3: aHR, 0.69 [95% CI, 0.61-0.78]; Q4: aHR, 0.75 [95% CI, 0.66-0.85]; *P* for trend < .001), and in women (Q2: aHR, 0.76 [95% CI, 0.69-0.84]; Q3: aHR, 0.73 [95% CI, 0.66-0.81]; Q4: aHR, 0.75 [95% CI, 0.67-0.84]; *P* for trend < .001).

**Table 2.** Risk of Disabling Dementia Risk According to Physical Activity in Men and Women

Physical activity	Men					Women				
	Q1	Q2	Q3	Q4	*P* for trend	Q1	Q2	Q3	Q4	*P* for trend
Daily total physical activity										
MET-h/d, median (range)	29.6 (22.4-32.1)	34.5 (32.1-37.5)	42.5 (37.6-49.0)	59.7 (49.0-92.5)	NA	29.9 (21.8-32.3)	34.5 (32.3-36.8)	39.8 (36.8-44.6)	52.9 (44.6-93.4)	NA
Person-years, No.	43 881	48 021	48 277	47 913	NA	53 221	57 776	58 623	59 314	NA
Incident dementia, No.	780	452	439	422	NA	1226	687	542	462	NA
Model 1 HR (95% CI)^a^	1 [Reference]	0.71 (0.63-0.80)	0.67 (0.60-0.76)	0.73 (0.65-0.83)	<.001	1 [Reference]	0.75 (0.68-0.83)	0.71 (0.64-0.79)	0.72 (0.64-0.80)	<.001
Model 1 HR (95% CI)^b^	1 [Reference]	0.73 (0.65-0.82)	0.69 (0.61-0.78)	0.75 (0.66-0.85)	<.001	1 [Reference]	0.76 (0.69-0.84)	0.73 (0.66-0.81)	0.75 (0.67-0.84)	<.001
Daily total MVPA										
MET-h/d, median (range)	0.4 (0-3.2)	6.0 (2.5-9.0)	16.7 (9.0-19.0)	42.0 (25.5-111.0)	NA	0.3 (0-2.5)	6.0 (2.5-9.0)	12.1 (9.0-19.0)	30.8 (19.1-108.0)	NA
Person-years, No.	44 019	48 076	48 147	47 852	NA	53 857	51 337	64 391	59 350	NA
Incident dementia, No.	768	449	455	421	NA	1152	650	655	460	NA
Model 1 HR (95% CI)^a^	1 [Reference]	0.72 (0.64-0.80)	0.65 (0.58-0.73)	0.72 (0.64-0.82)	<.001	1 [Reference]	0.81 (0.73-0.89)	0.74 (0.68-0.82)	0.71 (0.64-0.80)	<.001
Model 2 HR (95% CI)^b^	1 [Reference]	0.73 (0.65-0.82)	0.66 (0.59-0.74)	0.74 (0.65-0.84)	<.001	1 [Reference]	0.82 (0.74-0.91)	0.77 (0.69-0.85)	0.74 (0.66-0.83)	<.001
Leisure-time MVPA										
MET-h/d, median (range)	0	0.1 (0.03-0.3)	0.9 (0.3-1.6)	3.8 (1.6-58.5)	NA	0	0.1 (0.03-0.3)	0.8 (0.3-1.7)	3.8 (1.7-58.5)	NA
Person-years, No.	54 214	46 444	43 611	43 825	NA	76 689	50 942	49 662	51 642	NA
Incident dementia, No.	930	443	307	413	NA	1420	540	451	506	NA
Model 1 HR (95% CI)^a^	1 [Reference]	0.89 (0.79-1.00)	0.64 (0.56-0.73)	0.60 (0.53-0.67)	<.001	1 [Reference]	0.92 (0.83-1.02)	0.75 (0.67-0.83)	0.69 (0.62-0.76)	<.001
Model 2 HR (95% CI)^b^	1 [Reference]	0.90 (0.80-1.01)	0.65 (0.57-0.74)	0.59 (0.53-0.67)	<.001	1 [Reference]	0.94 (0.85-1.05)	0.76 (0.68-0.85)	0.70 (0.63-0.78)	<.001

Abbreviations: HR, hazard ratio; MET, metabolic equivalent; MVPA, moderate to vigorous physical activity; NA, not applicable; Q, quartile.

^a^ Adjusted for age and area.

^b^ Additionally adjusted for smoking status (never, former, 1-19 cigarettes/d, or ≥20 cigarettes/d), alcohol intake status (none or occasional drinkers, 1 to <150 g/week, 150 to <300 g/week, or ≥300 g/week), body mass index (calculated as weight in kilograms divided by height in meters squared; <18.5, 18.5-24.9, 25-29, or ≥30), past history of diabetes (yes or no), medication for hypertension (yes or no), and occupation (primary industry, secondary or tertiary industry, unemployed, or household duties).

Table 3 shows that an inverse association was present between daily total physical activity and risk of disabling dementia after excluding participants diagnosed within the first 3 and 6 years of the dementia ascertainment period in men and women, but that the associations were lost after exclusion of those diagnosed within the first 9 years of the dementia ascertainment period in both men (Q4 vs Q1: aHR, 0.99 [95% CI, 0.76-1.29]; *P* for trend = .69) and women (Q4 vs Q1: aHR, 0.93 [95% CI, 0.74-1.17]; *P* for trend = .51). Similar inverse associations were observed in men and women for total MVPA (men: aHR, 0.74 [95% CI, 0.65-0.84]; *P* for trend < .001; women: aHR, 0.74 [95% CI, 0.66-0.83]; *P* for trend < .001) and leisure-time MVPA (men: aHR, 0.59 [95% CI, 0.53-0.67]; *P* for trend < .001; women: aHR, 0.70 [95% CI, 0.63-0.78]; *P* for trend < .001). Regarding leisure-time MVPA, an inverse association with disabling dementia risk remained in men even after excluding participants diagnosed within first 9 years (Q4 vs Q1: aHR, 0.72 [95% CI, 0.56-0.92]; *P* for trend = .004), but not in women.

**Table 3.** Risk of Disabling Dementia Risk According to Physical Activity When Excluding Incident Disabling Dementia From the Starting Point

Physical activity	Men					Women				
	Q1	Q2	Q3	Q4	*P* for trend	Q1	Q2	Q3	Q4	*P* for trend
**Daily total physical activity**										
Excluding first 3 y										
Incident dementia, No.	570	356	364	333	NA	939	549	452	381	NA
Multivariable HR (95% CI)^a^	1 [Reference]	0.78 (0.68-0.89)	0.77 (0.67-0.88)	0.78 (0.68-0.91)	<.001	1 [Reference]	0.80 (0.72-0.89)	0.79 (0.70-0.89)	0.81 (0.71-0.92)	<.001
Excluding first 6 y										
Incident dementia, No.	379	238	248	239	NA	653	414	319	282	NA
Multivariable HR (95% CI)^a^	1 [Reference]	0.76 (0.65-0.90)	0.77 (0.65-0.90)	0.82 (0.69-0.98)	.02	1 [Reference]	0.88 (0.77-0.99)	0.78 (0.68-0.90)	0.84 (0.72-0.98)	.002
Excluding first 9 y										
Incident dementia, No.	151	103	100	114	NA	260	174	155	127	NA
Multivariable HR (95% CI)^a^	1 [Reference]	0.81 (0.63-1.05)	0.77 (0.59-1.00)	0.99 (0.76-1.29)	.69	1 [Reference]	0.92 (0.76-1.12)	0.94 (0.77-1.16)	0.93 (0.74-1.17)	.51
**Daily total MVPA**										
Excluding first 3 y										
Incident dementia, No.	566	351	374	332	NA	876	578	484	383	NA
Multivariable HR (95% CI)^a^	1 [Reference]	0.75 (0.65-0.86)	0.71 (0.62-0.81)	0.75 (0.65-0.87)	<.001	1 [Reference]	0.87 (0.78-0.97)	0.83 (0.74-0.93)	0.81 (0.71-0.92)	<.001
Excluding first 6 y										
Incident dementia, No.	377	235	252	240	NA	623	418	343	284	NA
Multivariable HR (95% CI)^a^	1 [Reference]	0.73 (0.62-0.85)	0.69 (0.59-0.82)	0.79 (0.67-0.94)	.002	1 [Reference]	0.87 (0.77-0.99)	0.79 (0.69-0.91)	0.81 (0.70-0.95)	.001
Excluding first 9 y										
Incident dementia, No.	147	101	107	113	NA	263	158	164	131	NA
Multivariable HR (95% CI)^a^	1 [Reference]	0.80 (0.62-1.04)	0.75 (0.59-0.97)	0.98 (0.76-1.27)	.58	1 [Reference]	0.80 (0.66-0.98)	0.86 (0.70-1.05)	0.87 (0.69-1.09)	.19
**Leisure-time MVPA**										
Excluding first 3 y										
No. of cases	695	351	243	334	NA	1085	430	393	413	NA
Multivariable HR (95% CI)^a^	1 [Reference]	0.93 (0.82-1.07)	0.67 (0.58-0.78)	0.63 (0.55-0.72)	<.001	1 [Reference]	0.97 (0.86-1.09)	0.85 (0.75-0.95)	0.73 (0.65-0.82)	<.001
Excluding first 6 y										
Incident dementia, No.	470	239	167	228	NA	754	303	297	314	NA
Multivariable HR (95% CI)^a^	1 [Reference]	0.92 (0.78-1.08)	0.66 (0.55-0.79)	0.61 (0.51-0.71)	<.001	1 [Reference]	0.97 (0.85-1.12)	0.90 (0.79-1.04)	0.78 (0.68-0.90)	<.001
Excluding first 9 y										
Incident dementia, No.	184	98	77	109	NA	290	132	145	149	NA
Multivariable HR (95% CI)^a^	1 [Reference]	0.90 (0.69-1.16)	0.73 (0.56-0.97)	0.72 (0.56-0.92)	.004	1 [Reference]	1.03 (0.83-1.28)	1.08 (0.88-1.32)	0.91 (0.75-1.12)	.59

Abbreviations: HR, hazard ratios; MVPA; moderate to vigorous physical activity; NA, not applicable; Q, quartile.

^a^ Adjusted for age, area, smoking status (never, former, 1-19 cigarettes/d, or ≥20 cigarettes/d), alcohol intake status (none or occasional drinkers, 1 to <150 g/week, 150 to <300 g/week, ≥300 g/week), body mass index (calculated as weight in kilograms divided by height in meters squared; <18.5, 18.5 to 24.9, 25 to 29, or ≥30), past history of diabetes (yes or no), medication for hypertension (yes or no), and occupation (primary industry, secondary or tertiary industry, unemployed or household duties).

Risks of disabling dementia in the highest vs lowest group of physical activity after excluding participants with disabling dementia diagnosed within the first 10 years of the dementia ascertainment period are shown in the Figure. The statistically significant associations between daily total physical activity and disabling dementia risk disappeared after excluding participants with disabling dementia diagnosed within the first 7 years in men (aHR, 0.93 [95%CI, 0.77-1.12]) and within the first 8 years in women (aHR, 0.86 [95%CI, 0.71-1.04]). In contrast, the inverse association between leisure-time MVPA and disabling dementia risk in men remained significant after excluding participants diagnosed within 10 years.

**Figure.**
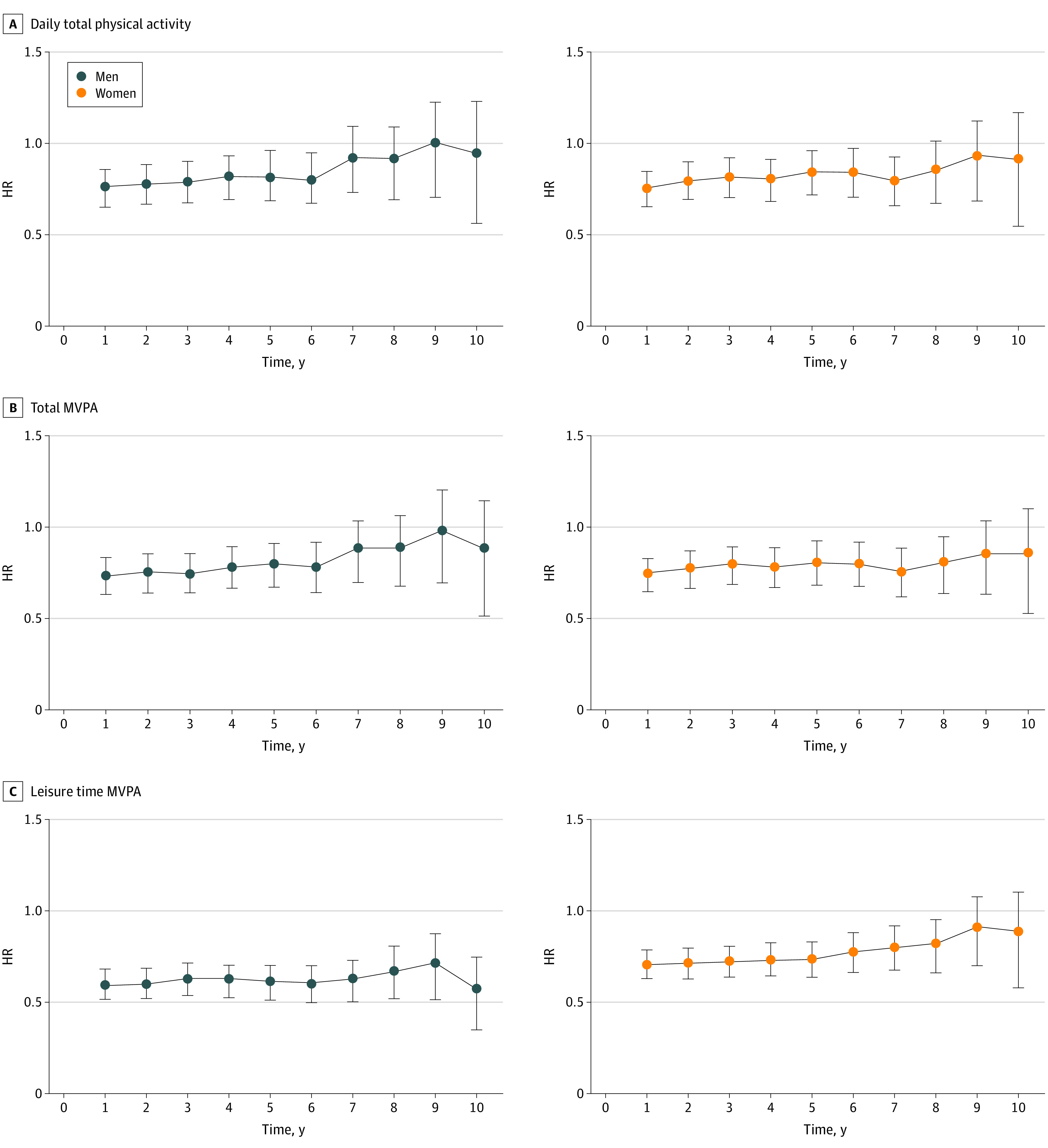
Risk of Disabling Dementia Risk in the Highest vs Lowest Groups of Physical Activity After Excluding Participants Diagnosed Within the First 10 Years of the Dementia Ascertainment Period HR indicates hazard ratio; MVPA, moderate to vigorous physical activity. Time was calculated as number of years from starting point during which participants disabling dementia were excluded from analysis. HRs were adjusted for age, area, smoking status, alcohol intake status, body mass index, past history of diabetes, medication for hypertension, and occupation.

We found no substantial difference in the associations of physical activity with disabling dementia in participants aged 65 years and older (eTable 1 in the Supplement). Regarding the analysis using death as a competing risk, no substantial differences were observed (eTable 2 in the Supplement). We found an increased risk of disabling dementia among participants who were in the lower tertiles of daily total physical activity in the 5-year questionnaire and the lower, middle, and upper tertiles in the 10-year questionnaire, as well as in those who were in the upper and middle tertiles of daily total physical activity in the 5-year questionnaire and the lower tertiles in the 10-year questionnaire (eTable 3 in the Supplement). Additionally, there were no substantial differences in the associations after addition of education level to the multivariable model in cohort I (eTable 4 in the Supplement).

## Discussion

In this prospective cohort study, we found that a higher level of physical activity was significantly associated with decreased risk of disabling dementia in men and women. However, after excluding participants diagnosed within 7 years from the starting point in men and within 8 years in women, the inverse associations of daily total physical activity and total MVPA with risk of disabling dementia incidence disappeared. These results suggest the potential presence of reverse causation bias. To our knowledge, this is the first prospective cohort study to suggest a potential for reverse causation bias between daily total physical activity and total MVPA and dementia. In contrast, a higher level of leisure-time MVPA was associated with reduced risk of disabling dementia in men.

Although 2 cohort studies^8,9^ have reported an inverse association between daily total physical activity and dementia risk, neither examined reverse causation bias. In the Honolulu-Asia Aging Study, Taaffe et al^8^ showed that a low level of daily total physical activity was associated with high risk of dementia in 173 patients with incident dementia with a mean follow-up of 6.1 years. In the Framingham study, Tan et al^9^ reported that a low level of daily total physical activity was associated with a higher risk of dementia in 236 patients with a longer follow-up of up to 22 years, but the study did not stratify results by sex owing to the low number of patients with incident dementia. To our knowledge, therefore, our study is the first large prospective study involving middle-aged and older participants with a longer dementia ascertainment period (9.5 years) and large number of incident diagnoses to observe potential reverse causation bias in the association between daily total physical activity and dementia risk. Given that 2 randomized clinical trials^30,31^ and a systematic review^32^ found that physical activity had little associations with improving cognitive function or the incidence of dementia, our results of no association between total physical activity and risk of disabling dementia after controlling for potential reverse causation bias may be plausible. One reason for potential reverse causation bias in this association was that daily total physical activity had already declined at the starting point in persons who would develop disabling dementia in the near future. In the preclinical phase of dementia, apathy is commonly observed as a neuropsychiatric feature,^33,34^ and persons with cognitive deficit^35^ and mild cognitive impairment (MCI)^36^ show features of behavioral inhibition.^37^ In fact, it has been reported that persons with MCI have a significantly lower physical activity level and a lower exercise capacity, which are associated with behavioral inhibition.^37^ Our results therefore suggest the presence of reverse causation bias in the association between daily total physical activity and risk of disabling dementia, as previously reported.^12^ Additionally, our results showing that a decline in daily total physical activity in the 5-year to 10-year questionnaire was positively associated with the risk of disabling dementia could also be considered as suggesting reverse causation bias. A further reason is that participants with a low level of physical activity may already have had other conditions that were likely to convert to or promote dementia or were associated with it.

Regarding the intensity of physical activity, a cohort study by Iso-Markku et al^38^ reported that the association of physical activity on cognitive function did not differ by the intensity of activity. This is consistent with our finding that the presence or disappearance of an association by exclusion of participants did not differ by the intensity of activity in both daily total physical activity and total MVPA.

In contrast, our results show that the linear trend of an inverse association between leisure-time MVPA and disabling dementia risk remained even after excluding participants diagnosed within the first 10 years in men. Therefore, a high level of physical activity in leisure-time may be associated with a decreased risk of disabling dementia in men. The findings of the Whitehall II cohort study^12^ suggested a reverse causation bias, namely that leisure-time physical activity was not associated with the risk of dementia in 329 participants and that preclinical decline in physical activity occurred in the 9 years before the diagnosis of dementia, while a meta-analysis by Kivimäki^15^ of 2044 participants with incident dementia also concluded that physical inactivity was not associated with all-cause dementia in men. An animal study by Barha et al^39^ reported that voluntary physical activity, such as in leisure-time, was associated with improving some cognitive functions, including memory, to a greater extent in males. Leisure-time MVPA measured in this study was derived from activities involving cognitive activity, such as golf and tennis.^28^ Leisure activities that include cognitive activity have a protective association against cognitive decline and dementia,^40^ and a randomized clinical trial reported that combined cognitive and exercise training could improve the cognitive functions of community-dwelling older adults.^41^ In addition, the social activity^42,43^ that accompanies leisure-time physical activities, such as participation in golf competitions and enrollment in tennis circles, also has a protective association against cognitive decline and dementia. The men in this study might therefore have been subject to different protective associations against disabling dementia through habitual leisure-time MVPA involving cognitive activity and social activity compared with men with less leisure-time MVPA. In contrast, this association of leisure-time MVPA may have been attenuated in women participants because women already engage in many cognitive activities through daily housework activities,^44^ and are likely to have a larger social network than men.^45^ Accordingly, our findings suggesting no reverse causation bias in the association between leisure-time MVPA and disabling dementia in men may be plausible.

The strengths of this study include its prospective design in participants aged 50 to 79 years at the survey point of exposure, long follow-up, large sample size, systematic registration of disabling dementia and comprehensive assessment of lifestyle factors, such as smoking status and alcohol consumption. These strengths allowed us to examine possible reverse causation bias in the association between daily total physical activity and disabling dementia, which few previous studies have investigated.

### Limitations

This study has several limitations. First, we could not assess the association between physical activity and specific types of dementia, such as Alzheimer disease and vascular dementia, because data from the LTCI system did not include type of dementia. In addition, several previous studies reported that type of dementia, such as Alzheimer disease, was not associated with mediating the association between physical activity and dementia risk.^9,11,18^ Accordingly, the risk of disabling dementia in our study participants was unlikely to have been associated with dementia type. Second, we could not completely eliminate misclassification in the diagnosis of disabling dementia conducted by attending physicians, albeit that a previous validation showed that such diagnosis had high specificity for the diagnosis of disabling dementia and was less likely to be affected by false-positive diagnoses.^23^ Third, we did not obtain information on education from all participants. We conducted a subanalysis of participants with a reported education level, but the results did not substantially differ when education level was included in the multivariable model. Fourth, the approach used to evaluate for potential reverse causation bias may have led to selection bias. Fifth, because we used information on physical activity at a single time point only (10-year follow-up survey), misclassification of exposure due to changes in physical activity during the dementia ascertainment period might have occurred. If present, however, misclassification of exposure would likely influence the true relative risk toward null.

## Conclusions

In this large prospective cohort study, we observed a potential reverse causation bias in the association of daily total physical activity and total MVPA with risk of disabling dementia in men and women. In contrast, however, leisure-time MVPA may have a protective association against disabling dementia in men.

## Notes

Supplement. **eFigure.** Flowchart of Study Participants **eTable 1.** Risk of Disabling Dementia Risk According to Physical Activity in Men and Women Aged 65 Years and Older **eTable 2.** Risk of Disabling Dementia Risk According to Physical Activity Using the Fine and Gray Subdistribution Hazards Model for Competing Risk of Death **eTable 3.** Risk of Disabling Dementia Risk According to the Change of Daily Total Physical Activity in Men and Women **eTable 4.** Risk of Disabling Dementia Risk According to Physical Activity in a Model Adding Education Level in Cohort I **eAppendix.** Members of the Japan Public Health Center-based Prospective Study (JPHC) Study Group
