# Supplementary material for: Association Between Physical Activity and Risk of Disabling Dementia in Japan
Source: JAMA Netw Open. 2022 Mar 29;5(3):e224590. doi: 10.1001/jamanetworkopen.2022.4590 (PMC8965633; doi:10.1001/jamanetworkopen.2022.4590)
Supplement: Supplement. — eFigure. Flowchart of Study Participants eTable 1. Risk of Disabling Dementia Risk According to Physical Activity in Men and Women Aged 65 Years and Older eTable 2. Risk of Disabling Dementia Risk According to Physical Activity Using the Fine and Gray Subdistribution Hazards Model for Competing Risk of Death eTable 3. Risk of Disabling Dementia Risk According to the Change of Daily Total Physical Activity in Men and Women eTable 4. Risk of Disabling Dementia Risk According to Physical Activity in a Model Adding Education Level in Cohort I eAppendix. Members of the Japan Public Health Center-based Prospective Study (JPHC) Study Group [file jamanetwopen-e224590-s001.pdf]

Supplemental Online Content

Ihira H, Sawada N, Inoue M, et al. Association between physical activity and risk of disabling dementia in Japan. *JAMA Netw Open*. 2022;5(3):e224590. doi:10.1001/jamanetworkopen.2022.4590

eFigure. Flowchart of Study Participants

eTable 1. Risk of Disabling Dementia Risk According to Physical Activity in Men and Women Aged 65 Years and Older

eTable 2. Risk of Disabling Dementia Risk According to Physical Activity Using the Fine and Gray Subdistribution Hazards Model for Competing Risk of Death

eTable 3. Risk of Disabling Dementia Risk According to the Change of Daily Total Physical Activity in Men and Women

eTable 4. Risk of Disabling Dementia Risk According to Physical Activity in a Model Adding Education Level in Cohort I

eAppendix. Members of the Japan Public Health Center-based Prospective Study (JPHC) Study Group

This supplemental material has been provided by the authors to give readers additional information about their work.

© 2022 Ihira H et al. *JAMA Network Open.*

eFigure. Flowchart of Study Participants

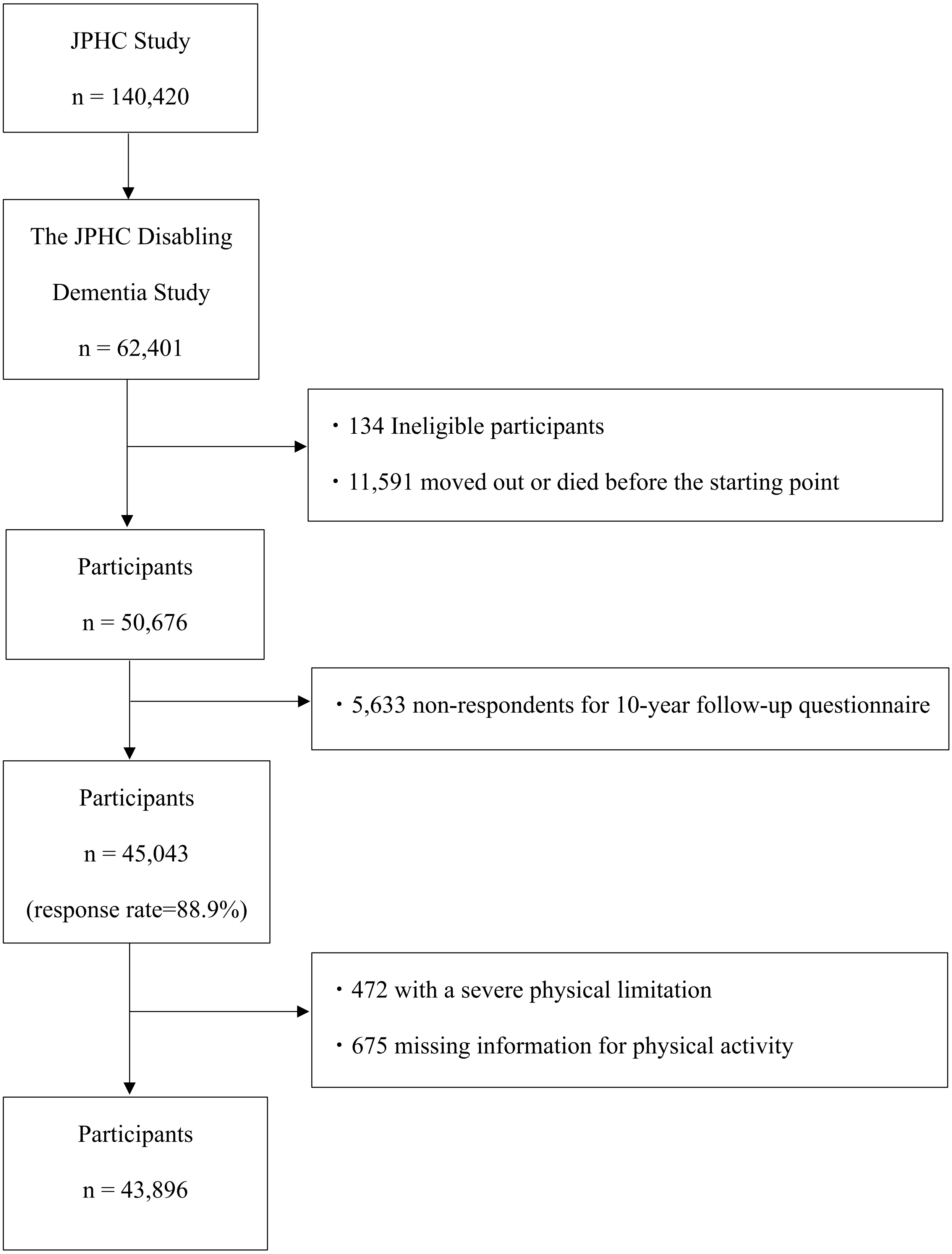

© 2022 Ihira H et al. *JAMA Network Open.*

eTable 1. Risk of Disabling Dementia Risk According to Physical Activity in Men and Women Aged 65 Years and Older

	Men					Women				
Physical activity	Q1	Q2	Q3	Q4	P-trend	Q1	Q2	Q3	Q4	P-trend
Daily total physical activity										
Median, MET-hours/day (range)	28.8 (22.4 to 30.4)	32.8 (30.4 to 35.8)	39.8 (35.8 to 45.6)	56.0 (45.6 to 92.5)		28.8 (21.8 to 30.3)	32.3 (30.4 to 34.3)	36.8 (34.3 to 40.6)	47.6 (40.6 to 93.4)	
Person-years	11387	13069	13613	13391		16023	17429	18410	18447	
Number of cases	465	364	321	314		731	593	474	455	
HR [a] (95%CI)	1	0.75 (0.65 to 0.86)	0.66 (0.57 to 0.77)	0.66 (0.57 to 0.76)	<.001	1	0.80 (0.71 to 0.89)	0.67 (0.59 to 0.75)	0.69 (0.61 to 0.78)	<.001
HR [b] (95%CI)	1	0.76 (0.66 to 0.87)	0.67 (0.58 to 0.78)	0.66 (0.57 to 0.77)	<.001	1	0.80 (0.72 to 0.89)	0.68 (0.60 to 0.76)	0.71 (0.63 to 0.81)	<.001
Excluding first 3 years										

© 2022 Ihira H et al. *JAMA Network Open.*

Number of cases	334	276	252	247		550	473	377	367	
HR [b] (95%CI)	1	0.80 (0.68 to 0.94)	0.74 (0.62 to 0.87)	0.72 (0.60 to 0.85)	<.001	1	0.88 (0.78 to 1.00)	0.73 (0.64 to 0.84)	0.78 (0.68 to 0.90)	<.001
Excluding first 6 years										
Number of cases	219	179	168	170		386	333	274	259	
HR [b] (95%CI)	1	0.78 (0.64 to 0.95)	0.74 (0.60 to 0.91)	0.75 (0.61 to 0.93)	.007	1	0.87 (0.75 to 1.01)	0.74 (0.63 to 0.87)	0.76 (0.64 to 0.90)	<.001
Excluding first 9 years										
Number of cases	91	60	68	74		152	126	124	110	
HR [b] (95%CI)	1	0.60 (0.43 to 0.83)	0.72 (0.52 to 0.99)	0.79 (0.57 to 1.08)	.23	1	0.84 (0.66 to 1.06)	0.87 (0.68 to 1.12)	0.82 (0.63 to 1.06)	.17
Daily total MVPA										
Median, MET-hours/day (range)	0 (0 to 1.4)	4.5 (1.5 to 9.0)	13.6 (9.0 to 22.2)	36.0 (22.5 to 111.0)		0 (0 to 1.3)	2.0 (1.5 to 6.0)	9.0 (6.0 to 14.9)	24.1 (15.0 to 104.8)	
Person-years	9957	14148	13930	13426		14932	15279	21451	18647	

© 2022 Ihira H et al. *JAMA Network Open.*

Number of cases	412	408	330	314		672	528	580	473	
HR [a] (95%CI)	1	0.71 (0.62 to 0.82)	0.64 (0.55 to 0.74)	0.64 (0.55 to 0.74)	<.001	1	0.82 (0.73 to 0.92)	0.69 (0.62 to 0.78)	0.71 (0.63 to 0.80)	<.001
HR [b] (95%CI)	1	0.73 (0.63 to 0.84)	0.65 (0.56 to 0.76)	0.65 (0.55 to 0.75)	<.001	1	0.82 (0.73 to 0.92)	0.70 (0.62 to 0.78)	0.74 (0.65 to 0.83)	<.001
Excluding first 3 years										
Number of cases	339	253	270	247		501	501	377	388	
HR [b] (95%CI)	1	0.73 (0.62 to 0.86)	0.72 (0.61 to 0.85)	0.70 (0.59 to 0.83)	<.001	1	0.83 (0.73 to 0.94)	0.72 (0.63 to 0.83)	0.78 (0.68 to 0.90)	<.001
Excluding first 6 years										
Number of cases	227	172	166	171		344	353	284	271	
HR [b] (95%CI)	1	0.67 (0.55 to 0.82)	0.67 (0.54 to 0.82)	0.70 (0.57 to 0.87)	.001	1	0.84 (0.72 to 0.97)	0.72 (0.62 to 0.85)	0.78 (0.66 to 0.92)	.001
Excluding first 9 years										
Number of cases	84	69	66	74		135	133	127	117	

© 2022 Ihira H et al. *JAMA Network Open.*

HR [b] (95%CI)	1	0.71 (0.51 to 0.98)	0.73 (0.52 to 1.01)	0.82 (0.60 to 1.13)	.26	1	0.73 (0.57 to 0.94)	0.83 (0.65 to 1.07)	0.82 (0.63 to 1.07)	.28
Leisure-time MVPA										
Median, MET-hours/day (range)	0	0.1 (0.03 to 0.4)	1.0 (0.4 to 2.3)	4.8 (2.3 to 58.5)		0	0.1 (0.03 to 0.3)	1.0 (0.3 to 2.3)	4.5(2.3 to 31.5)	
Person-years	18841	10734	10573	11313		29527	13814	14307	12662	
Number of cases	673	313	231	247		1126	442	361	324	
HR [a] (95%CI)	1	0.94 (0.82 to 1.08)	0.67 (0.57 to 0.77)	0.66 (0.57 to 0.77)	<.001	1	0.93 (0.83 to 1.04)	0.72 (0.63 to 0.81)	0.76 (0.67 to 0.86)	<.001
HR [b] (95%CI)	1	0.95 (0.82 to 1.09)	0.66 (0.57 to 0.77)	0.65 (0.56 to 0.75)	<.001	1	0.95 (0.84 to 1.06)	0.72 (0.64 to 0.81)	0.77 (0.68 to 0.87)	<.001
Excluding first 3 years										
Number of cases	490	243	183	193		853	342	311	261	
HR [b] (95%CI)	1	0.99 (0.85 to 1.17)	0.69 (0.58 to 0.82)	0.70 (0.59 to 0.83)	<.001	1	0.95 (0.83 to 1.08)	0.80 (0.70 to 0.91)	0.79 (0.69 to 0.91)	<.001

© 2022 Ihira H et al. *JAMA Network Open.*

Excluding first 6 years										
Number of cases	326	156	122	132		589	235	231	197	
HR [b] (95%CI)	1	0.95 (0.78 to 1.16)	0.66 (0.53 to 0.82)	0.68 (0.56 to 0.84)	<.001	1	0.93 (0.80 to 1.09)	0.84 (0.72 to 0.98)	0.84 (0.71 to 0.99)	.01
Excluding first 9 years										
Number of cases	125	60	48	60		217	97	115	83	
HR [b] (95%CI)	1	0.90 (0.66 to 1.24)	0.67 (0.47 to 0.95)	0.79 (0.57 to 1.09)	.05	1	0.99 (0.77 to 1.27)	1.09 (0.87 to 1.37)	0.93 (0.71 to 1.20)	.89

MET, metabolic equivalent; Q, quartile; HR, hazard ratio; CI, confidence interval; MVPA; moderate to vigorous physical activity

[a] Adjusted for age and area

[b] Additionally adjusted for smoking status (never, former, 1 to 19 cigarettes/day, ≥20 cigarettes/day) and alcohol intake status (none or occasional drinkers, 1 to <150g/week, 150 to <300g/week, ≥ 300g/week), body mass index (<18.5, 18.5 to 24.9, 25 to 29, ≥30), past history of diabetes (yes, no), medication for hypertension (yes, no), and occupation (primary industry, secondary or tertiary industry, unemployed, household duties).

© 2022 Ihira H et al. *JAMA Network Open.*

eTable 2. Risk of Disabling Dementia Risk According to Physical Activity Using the Fine and Gray Subdistribution Hazards Model for Competing Risk of Death

	Men					Women				
Physical activity	Q1	Q2	Q3	Q4	P-trend	Q1	Q2	Q3	Q4	P-trend
Daily total physical activity										
Median, MET-hours/day (range)	29.6 (22.4 to 32.1)	34.5 (32.1 to 37.5)	42.5 (37.6 to 49.0)	59.7 (49.0 to 92.5)		29.9 (21.8 to 32.3)	34.5 (32.3 to 36.8)	39.8 (36.8 to 44.6)	52.9 (44.6 to 93.4)	
Person-years	43881	48021	48277	47913		53221	57776	58623	59314	
Number of cases	780	452	439	422		1226	687	542	462	
HR [a] (95%CI)	1	0.78 (0.69 to 0.88)	0.74 (0.65 to 0.83)	0.79 (0.70 to 0.89)	<.001	1	0.79 (0.71 to 0.86)	0.74 (0.67 to 0.82)	0.75 (0.67 to 0.85)	<.001
HR [b] (95%CI)	1	0.81 (0.72 to 0.91)	0.76 (0.68 to 0.86)	0.81 (0.71 to 0.92)	<.001	1	0.82 (0.75 to 0.91)	0.78 (0.70 to 0.87)	0.80 (0.71 to 0.90)	<.001
Excluding first 3 years										
Number of cases	570	356	364	333		939	549	452	381	

© 2022 Ihira H et al. *JAMA Network Open.*

HR [b] (95%CI)	1	0.88 (0.76 to 1.00)	0.87 (0.75 to 0.99)	0.87 (0.75 to 1.00)	.04	1	0.86 (0.77 to 0.96)	0.85 (0.76 to 0.96)	0.86 (0.75 to 0.98)	0.008
Excluding first 6 years										
Number of cases	379	238	248	239		653	414	319	282	
HR [b] (95%CI)	1	0.87 (0.74 to 1.03)	0.88 (0.74 to 1.04)	0.92 (0.78 to 1.10)	.30	1	0.95 (0.84 to 1.08)	0.86 (0.74 to 0.98)	0.90 (0.77 to 1.05)	.05
Excluding first 9 years										
Number of cases	151	103	100	114		260	174	155	127	
HR [b] (95%CI)	1	0.95 (0.73 to 1.22)	0.90 (0.69 to 1.17)	1.12 (0.86 to 1.46)	.60	1	0.99 (0.81 to 1.21)	1.03 (0.84 to 1.27)	1.00 (0.79 to 1.26)	.91
Daily total MVPA										
Median, MET-hours/day (range)	0.4 (0 to 3.2)	6.0 (2.5 to 9.0)	16.7 (9.0 to 19.0)	42.0 (25.5 to 111.0)		0.3 (0 to 2.5)	6.0 (2.5 to 9.0)	12.1 (9.0 to 19.0)	30.8 (19.1 to 108.0)	
Person-years	44019	48076	48147	47852		53857	51337	64391	59350	
Number of cases	768	449	455	421		1152	650	655	460	

© 2022 Ihira H et al. *JAMA Network Open.*

HR [a] (95%CI)	1	0.78 (0.70 to 0.88)	0.71 (0.63 to 0.80)	0.78 (0.69 to 0.88)	<.001	1	0.84 (0.77 to 0.93)	0.78 (0.71 to 0.86)	0.75 (0.67 to 0.84)	<.001
HR [b] (95%CI)	1	0.81 (0.71 to 0.91)	0.72 (0.64 to 0.82)	0.79 (0.70 to 0.90)	<.001	1	0.89 (0.80 to 0.98)	0.83 (0.75 to 0.92)	0.79 (0.70 to 0.89)	<.001
Excluding first 3 years										
Number of cases	566	351	374	332		876	578	484	383	
HR [b] (95%CI)	1	0.84 (0.73 to 0.97)	0.80 (0.70 to 0.91)	0.83 (0.71 to 0.95)	.003	1	0.93 (0.83 to 1.03)	0.90 (0.80 to 1.01)	0.86 (0.75 to 0.98)	0.02
Excluding first 6 years										
Number of cases	377	235	252	240		623	418	343	284	
HR [b] (95%CI)	1	0.83 (0.70 to 0.97)	0.79 (0.67 to 0.93)	0.88 (0.74 to 1.04)	.06	1	0.94 (0.82 to 1.06)	0.87 (0.75 to 0.99)	0.87 (0.75 to 1.01)	.03
Excluding first 9 years										
Number of cases	147	101	107	113		263	158	164	131	

© 2022 Ihira H et al. *JAMA Network Open.*

HR [b] (95%CI)	1	0.94 (0.73 to 1.22)	0.88 (0.68 to 1.13)	1.11 (0.85 to 1.44)	.70	1	0.86 (0.70 to 1.05)	0.94 (0.76 to 1.15)	0.94 (0.75 to 1.17)	.60
Leisure-time MVPA										
Median, MET-hours/day (range)	0	0.1 (0.03 to 0.3)	0.9 (0.3 to 1.6)	3.8 (1.6 to 58.5)		0	0.1 (0.03 to 0.3)	0.8 (0.3 to 1.7)	3.8 (1.7 to 58.5)	
Person-years	54214	46444	43611	43825		76689	50942	49662	51642	
Number of cases	930	443	307	413		1420	540	451	506	
HR [a] (95%CI)	1	0.92 (0.82 to 1.03)	0.67 (0.59 to 0.76)	0.65 (0.58 to 0.73)	<.001	1	0.91 (0.82 to 1.01)	0.78 (0.71 to 0.87)	0.71 (0.64 to 0.79)	<.001
HR [b] (95%CI)	1	0.95 (0.84 to 1.07)	0.69 (0.61 to 0.79)	0.66 (0.59 to 0.75)	<.001	1	0.96 (0.86 to 1.07)	0.83 (0.74 to 0.92)	0.75 (0.67 to 0.83)	<.001
Excluding first 3 years										
Number of cases	695	351	243	334		1085	430	393	413	
HR [b] (95%CI)	1	0.98 (0.86 to 1.12)	0.72 (0.62 to 0.83)	0.71 (0.62 to 0.81)	<.001	1	0.97 (0.86 to 1.09)	0.92 (0.81 to 1.03)	0.77 (0.69 to 0.87)	<.001

© 2022 Ihira H et al. *JAMA Network Open.*

Excluding first 6 years										
Number of cases	470	239	167	228		754	303	297	314	
HR [b] (95%CI)	1	0.96 (0.81 to 1.13)	0.70 (0.58 to 0.84)	0.69 (0.58 to 0.81)	<.001	1	0.97 (0.84 to 1.12)	0.99 (0.86 to 1.13)	0.83 (0.73 to 0.96)	0.02
Excluding first 9 years										
Number of cases	184	98	77	109		290	132	145	149	
HR [b] (95%CI)	1	0.94 (0.73 to 1.23)	0.81 (0.61 to 1.07)	0.84 (0.66 to 1.08)	.11	1	1.04 (0.84 to 1.29)	1.19 (0.97 to 1.47)	0.98 (0.80 to 1.21)	.73

MET, metabolic equivalent; Q, quartile; HR, hazard ratio; CI, confidence interval; MVPA; moderate to vigorous physical activity

[a] Adjusted for age and area

[b] Additionally adjusted for smoking status (never, former, 1 to 19 cigarettes/day, ≥20 cigarettes/day) and alcohol intake status (none or occasional drinkers, 1 to <150g/week, 150 to <300g/week, ≥ 300g/week), body mass index (<18.5, 18.5 to 24.9, 25 to 29, ≥30) and past history of diabetes (yes, no), medication for hypertension (yes, no), occupation (primary industry, secondary or tertiary industry, unemployed, household duties).

© 2022 Ihira H et al. *JAMA Network Open.*

eTable 3. Risk of Disabling Dementia Risk According to the Change of Daily Total Physical Activity in Men and Women

Men									
Change in daily total physical activity	Q05G3–Q10G3	Q05G3–Q10G2	Q05G3–Q10G1	Q05G2–Q10G3	Q05G2–Q10G2	Q05G2–Q10G1	Q05G1–Q10G3	Q05G1–Q10G2	Q05G1–Q10G1
Person-years	30842	20562	13341	17296	23412	21040	7438	11932	17765
Number of cases	230	183	244	165	202	303	82	124	256
HR [a] (95%CI)	1	0.98 (0.81 to 1.19)	1.64 (1.37 to 1.97)	1.35 (1.10 to 1.64)	1.16 (0.96 to 1.41)	1.40 (1.18 to 1.67)	1.51 (1.17 to 1.95)	1.45 (1.16 to 1.80)	1.58 (1.31 to 1.89)
HR [b] (95%CI)	1	0.99 (0.82 to 1.21)	1.61 (1.33 to 1.93)	1.31 (1.07 to 1.61)	1.17 (0.97 to 1.42)	1.38 (1.16 to 1.66)	1.48 (1.15 to 1.91)	1.43 (1.15 to 1.79)	1.55 (1.29 to 1.87)
Women									
Change in daily total physical activity	Q05G3–Q10G3	Q05G3–Q10G2	Q05G3–Q10G1	Q05G2–Q10G3	Q05G2–Q10G2	Q05G2–Q10G1	Q05G1–Q10G3	Q05G1–Q10G2	Q05G1–Q10G1
Person-years	28996	20680	12982	16844	21735	23197	18355	26777	33835
Number of cases	643	221	139	315	226	178	350	244	229

© 2022 Ihira H et al. *JAMA Network Open.*

HR [a] (95%CI)	1	1.04 (0.87 to 1.25)	1.46 (1.23 to 1.73)	1.10 (0.90 to 1.34)	1.13 (0.94 to 1.35)	1.30 (1.09 to 1.54)	1.37 (1.11 to 1.69)	1.25 (1.04 to 1.51)	1.61 (1.38 to 1.88)
HR [b] (95%CI)	1	1.06 (0.89 to 1.28)	1.43 (1.21 to 1.70)	1.12 (0.92 to 1.36)	1.13 (0.94 to 1.36)	1.25 (1.05 to 1.49)	1.38 (1.11 to 1.70)	1.24 (1.03 to 1.50)	1.55 (1.32 to 1.81)
HR, hazard ratios; CI, confidence interval; Q05, 5-year questionnaire; Q10, 10-year questionnaire									
Q05: G1 <27.1; G2 27.1-34.3 G3 ≥34.3, Q10: G1 <33.8 ; G2 33.8-44.1; G3 ≥44.1 for men									
Q05: G1 <28.3 ; G2 28.3-34.3 ; G3 ≥34.3, Q10: G1 <33.8; G2 33.8–41.4; G3 ≥41.4 for women									
[a] Adjusted for age and area									
[b] Adjusted for age, area, smoking status (never, former, 1 to 19 cigarettes/day, ≥20 cigarettes/day), alcohol intake status (none or occasional drinkers, 1 to <150g/week, 150 to <300g/week, ≥ 300g/week), body mass index (<18.5, 18.5 to 24.9, 25 to 29, ≥30), past history of diabetes (yes, no), medication for hypertension (yes, no), and occupation (primary industry, secondary or tertiary industry, unemployed, household duties).									

© 2022 Ihira H et al. *JAMA Network Open.*

eTable 4. Risk of Disabling Dementia Risk According to Physical Activity in a Model Adding Education Level in Cohort I

	Men					Women				
Physical activity	Q1	Q2	Q3	Q4	P-trend	Q1	Q2	Q3	Q4	P-trend
Daily total physical activity										
Median (MET-hours/day)	29.7	35.0	43.6	60.9		30.1	34.7	40.0	52.9	
Person-years	21898	23186	23152	22654		27469	28289	28305	28694	
Number of cases	314	194	202	192		400	306	257	210	
HR [a] (95%CI)	1	0.68 (0.57 to 0.82)	0.67 (0.56 to 0.80)	0.74 (0.61 to 0.88)	< .001	1	0.87 (0.75 to 1.01)	0.78 (0.67 to 0.92)	0.76 (0.64 to 0.90)	< .001
HR [b] (95%CI)	1	0.75 (0.62 to 0.90)	0.73 (0.61 to 0.87)	0.77 (0.63 to 0.93)	.003	1	0.94 (0.79 to 1.11)	0.86 (0.72 to 1.03)	0.82 (0.67 to 1.01)	.03
Excluding first 3 years										
Number of cases	229	159	166	145		311	246	208	168	

© 2022 Ihira H et al. *JAMA Network Open.*

HR b (95%CI)	1	0.85 (0.69 to 1.05)	0.82 (0.67 to 1.01)	0.78 (0.63 to 0.98)	.03	1	0.94 (0.79 to 1.11)	0.86 (0.72 to 1.03)	0.82 (0.67 to 1.01)	.11
Excluding first 6 years										
Number of cases	161	96	117	103		224	182	147	126	
HR b (95%CI)	1	0.73 (0.56 to 0.94)	0.82 (0.64 to 1.05)	0.78 (0.60 to 1.02)	.10	1	0.97 (0.79 to 1.18)	0.85 (0.69 to 1.05)	0.86 (0.68 to 1.09)	.11
Excluding first 9 years										
Number of cases	64	40	50	47		87	70	65	63	
HR b (95%CI)	1	0.76 (0.51 to 1.13)	0.91 (0.62 to 1.33)	0.92 (0.62 to 1.38)	.81	1	0.93 (0.68 to 1.28)	0.97 (0.70 to 1.35)	1.12 (0.79 to 1.60)	.58
Total MVPA										
Median (MET-hours/day)	0.5	6.4	18.0	42.5		0.3	6.0	12.1	30.7	
Person-years	21921	23137	23168	22666		27796	24655	31645	28661	
Number of cases	315	182	208	197		389	265	314	205	

© 2022 Ihira H et al. *JAMA Network Open.*

HR [a] (95%CI)	1	0.65 (0.54 to 0.78)	0.64 (0.54 to 0.76)	0.74 (0.62 to 0.88)	< .001	1	0.85 (0.73 to 1.00)	0.81 (0.70 to 0.94)	0.71 (0.60 to 0.84)	< .001
HR [b] (95%CI)	1	0.70 (0.58 to 0.84)	0.69 (0.57 to 0.82)	0.77 (0.64 to 0.93)	.002	1	0.87 (0.74 to 1.02)	0.85 (0.73 to 0.99)	0.74 (0.62 to 0.88)	.001
Excluding first 3 years										
Number of cases	232	149	166	152		305	210	251	167	
HR [b] (95%CI)	1	0.77 (0.62 to 0.95)	0.73 (0.59 to 0.89)	0.78 (0.63 to 0.97)	.01	1	0.90 (0.75 to 1.08)	0.87 (0.73 to 1.03)	0.77 (0.63 to 0.94)	.01
Excluding first 6 years										
Number of cases	159	94	118	106		220	152	183	124	
HR [b] (95%CI)	1	0.69 (0.54 to 0.90)	0.74 (0.58 to 0.94)	0.78 (0.60 to 1.01)	.05	1	0.90 (0.73 to 1.11)	0.87 (0.72 to 1.06)	0.79 (0.62 to 0.99)	.04
Excluding first 9 years										
Number of cases	63	39	49	50		87	72	63	63	

© 2022 Ihira H et al. *JAMA Network Open.*

HR [b] (95%CI)	1	0.71 (0.47 to 1.05)	0.80 (0.54 to 1.17)	0.94 (0.63 to 1.39)	.75	1	0.91 (0.66 to 1.24)	0.86 (0.62 to 1.20)	1.04 (0.73 to 1.46)	.97
Leisure-time MVPA										
Median (MET-hours/day)	0	0.2	1.1	4.5		0	0.2	1.1	4.5	
Person-years	34114	19211	20014	17552		49053	21809	22000	19895	
Number of cases	450	147	159	146		632	178	175	188	
HR [a] (95%CI)	1	0.72 (0.60 to 0.87)	0.61 (0.50 to 0.73)	0.54 (0.45 to 0.65)	< .001	1	0.79 (0.67 to 0.93)	0.68 (0.57 to 0.80)	0.73 (0.62 to 0.86)	< .001
HR [b] (95%CI)	1	0.76 (0.63 to 0.92)	0.65 (0.54 to 0.79)	0.57 (0.46 to 0.69)	< .001	1	0.81 (0.69 to 0.97)	0.70 (0.59 to 0.83)	0.75 (0.63 to 0.89)	< .001
Excluding first 3 years										
Number of cases	344	111	128	116		487	145	153	148	
HR [b] (95%CI)	1	0.74 (0.60 to 0.92)	0.70 (0.56 to 0.86)	0.58 (0.47 to 0.73)	< .001	1	0.86 (0.71 to 1.04)	0.80 (0.66 to 0.96)	0.76 (0.63 to 0.92)	.001

© 2022 Ihira H et al. *JAMA Network Open.*

Excluding first 6 years										
Number of cases	233	78	88	78		351	103	115	110	
HR [b] (95%CI)	1	0.75 (0.58 to 0.98)	0.69 (0.54 to 0.89)	0.56 (0.42 to 0.73)	< .001	1	0.84 (0.68 to 1.06)	0.82 (0.66 to 1.02)	0.78 (0.62 to 0.97)	.01
Excluding first 9 years										
Number of cases	86	42	38	35		135	43	56	51	
HR [b] (95%CI)	1	1.12 (0.76 to 1.64)	0.82 (0.55 to 1.23)	0.69 (0.46 to 1.04)	.05	1	0.88 (0.63 to 1.25)	1.01 (0.74 to 1.39)	0.89 (0.64 to 1.24)	.59

MET, metabolic equivalent; Q, quartile; HR, hazard ratio; CI, confidence interval; MVPA; moderate to vigorous physical activity

[a] Adjusted for age and area

[b] Additionally adjusted for smoking status (never, former, 1 to 19 cigarettes/day, ≥20 cigarettes/day) and alcohol intake status (none or occasional drinkers, 1 to <150g/week, 150 to <300g/week, ≥ 300g/week), body mass index (<18.5, 18.5 to 24.9, 25 to 29, ≥30), past history of diabetes (yes, no), medication for hypertension (yes, no), occupation (primary industry, secondary or tertiary industry, unemployed, household duties) and education level (junior high school, higher education).

© 2022 Ihira H et al. *JAMA Network Open.*

eAppendix. Members of the JPHC Study Group

Members of the JPHC Study Group are listed in https://epi.ncc.go.jp/en/jphc/781/8390.html

© 2022 Ihira H et al. *JAMA Network Open.*
